# Geostatistical Analysis of White Matter Lesions in Multiple Sclerosis Identifies Gender Differences in Lesion Evolution

**DOI:** 10.3389/fnmol.2018.00460

**Published:** 2018-12-17

**Authors:** Robert Marschallinger, Mark Mühlau, Viola Pongratz, Jan S. Kirschke, Simon Marschallinger, Paul Schmidt, Johann Sellner

**Affiliations:** ^1^Geoinformatics Z_GIS, University of Salzburg, Salzburg, Austria; ^2^Department of Neurology, Christian Doppler Medical Center, Paracelsus Medical University, Salzburg, Austria; ^3^Department of Neurology, Klinikum Rechts der Isar, Technische Universität München, Munich, Germany; ^4^TUM Neuroimaging Center, Klinikum Rechts der Isar, Technische Universität München, Munich, Germany; ^5^Institute of Neuroradiology, Klinikum Rechts der Isar, Technische Universität München, Munich, Germany; ^6^Fachhochschule für Gesundheitsberufe Oberösterreich GmbH, Linz, Austria

**Keywords:** multiple Sclerosis, geostatistics, 4D analysis, lesion segmentation, white matter lesion evolution, gender differences, time series analysis

## Abstract

Multiple sclerosis (MS) is a chronic inflammatory demyelinating disease of the central nervous system with presumed autoimmune origin. The development of lesions within the gray matter and white matter, which are highly variable with respect to number, total volume, morphology and spatial evolution and which only show a limited correlation with clinical disability, is a hallmark of the disease. Population-based studies indicate a distinct outcome depending on gender. Here, we studied gender-related differences in the evolution of white matter MS-lesions (MS-WML) in early MS by using geostatistical methods. Within a 3 years observation period, a female and a male MS patient group received disease modifying drugs and underwent standardized annual brain magnetic resonance imaging, accompanied by neurological examination. MS-WML were automatically extracted and the derived binary lesion masks were subject to geostatistical analysis, yielding quantitative spatial-statistics metrics on MS-WML pattern morphology and total lesion volume (TLV). Through the MS-lesion pattern discrimination plot, the following differences were disclosed: corresponding to gender and MS-WML pattern morphology at baseline, two female subgroups (F1, F2) and two male subgroups (M1, M2) are discerned that follow a distinct MS-WML pattern evolution in space and time. F1 and M1 start with medium-level MS-WML pattern smoothness and TLV, both behave longitudinally quasi-static. By contrast, F2 and M2 start with high-level MS-WML pattern smoothness and medium-level TLV. F2 and M2 longitudinal development is characterized by strongly diminishing MS-WML pattern smoothness and TLV, i.e., continued shrinking and break-up of MS-WML. As compared to the male subgroup M2, the female subgroup F2 shows continued, increased MS-WML pattern smoothness and TLV. Data from neurological examination suggest a correlation of MS-WML pattern morphology metrics and EDSS. Our results justify detailed studies on gender-related differences.

## Introduction

Multiple sclerosis (MS) is an inflammatory and neurodegenerative autoimmune disease of the central nervous system (CNS), characterized by demyelination and axonal degeneration. Magnetic resonance imaging (MRI) plays a central role in the diagnosis and management of patients with MS: focal hyperintense lesions seen on T2 and FLAIR MRI sequences throughout the CNS are key features of the disorder. MRI is the key tool to assess dissemination in space (DIS) and dissemination in time (DIT) (Thompson et al., 2018). Moreover, MRI is used to monitor disease activity and to evaluate therapeutic responses. Yet, changes in T2 lesion volume are poor predictors of subsequent disease evolution in many cases, a situation often referred to as the “clinicoradiological paradox” (Louapre, 2018). The heterogeneity of the disease may also be impacted by gender differences: while the incidence of MS is consistently greater in females, men have been reported to develop a more severe disease phenotype characterized by a worse clinical outcome and faster accumulation of disability (Tomassini and Pozzilli, 2009; Shirani et al., 2012). Gender, however, does not influence relapse and burden of brain lesions (Dolezal et al., 2013; Harbo et al., 2013; Kalincik et al., 2013). In this regard, Schoonheim (2014) studied gender differences in brain MRI using diffusion tensor imaging, which enables the evaluation of white matter (WM) tracts. They found that WM of male patients was both more extensively and also more severely affected compared to female patients. A combined usage of novel imaging techniques and spatial-statistics models may provide further insights to the pathophysiological background of gender differences in disease course and lesion evolution. Based on geostatistical metrics (Marschallinger et al., 2016), we established a methodology to study MS-WML pattern characteristics including lesion pattern morphology. Here, we tested our geostatistical methodology in a longitudinal group consisting of men and women in order to evaluate gender-related changes and differences in MS-WML evolution.

## Materials and Methods

### Patient Groups

To study the spatiotemporal evolution of MS-WML patterns, we evaluated two groups of early-stage MS patients: groups consist of women (“F”: *n* = 53) and men (“M”: *n* = 36), respectively. The association of WML with MS was checked by an experienced neuroradiologist. During the 3-year study period, F and M group members received disease modifying drug (DMD) and underwent four MRI investigations (MRI1, MRI2, MRI3, MRI4) under standardized conditions: identical 3T MRI scanner, identical protocols (see below) and 1 year time interval between each MRI1-MRI2-MRI3-MRI4. Additional criteria for F and M group inclusion were: time from first reported MS symptoms to MRI1 less than 2 years and time from MS-diagnosis to MRI1 less than 1 year. Members of both groups were checked for EDSS at all times of MRI scans and for disease course at MRI1, MRI2, and MRI4. Two-sample *t*-test and Fisher *F*-test indicate equal means and variances (α = 0.05) of groups F and M; we therefore assume an identical age structure of both groups. Table 1 summarizes group ages, MRI investigation intervals and the development of clinical status in the time interval from MRI1 to MRI4. Average investigation interval in both groups was about 1 year ± 1 month. Average EDSS for groups F and M increased slightly. Both groups started with a mix of clinically isolated syndrome (CIS) and relapsing-remitting MS (RRMS). As indicated by Table 1, both groups were affected by marked shifts in disease type, from CIS to RRMS.

**Table 1:** Descriptive statistics of ages, MRI acquisition intervals and clinical parameters of groups F and M.

	*n*	Age(y)		Lag(d)		EDSS1		EDSS4		DType1(%)		DType4(%)	
		avg	std	avg	std	avg	std	avg	std	CIS	RRMS	CIS	RRMS
F	53	33,3	9,0	369	32	1,3	1,0	1,4	1,4	58	42	6	94
M	36	35,1	9,7	365	31	1,2	1,2	1,3	1,3	50	50	14	86

*n: number of group members. Age(y): average (avg) and standard deviation (std) of group ages, years. Lag(d): average and standard deviation between MRI1-MRI2, MRI2-MRI3, MRI3-MRI4, days. EDSS1, EDSS4: group average and standard deviation of EDSS at MRI1 and MRI4. DType1, DType4: disease course of group members at MRI1 and MRI4, per cent.*

### MRI Protocols

All subjects underwent MRI scanning at the identical 3T scanner (Philips Achieva) using identical protocols. A 3D gradient echo T1w sequence was acquired using magnetization-prepared 180 degrees radiofrequency pulses and rapid gradient-echo sampling with a spatial resolution of 1.0 mm × 1.0 mm × 1.0 mm, a repetition time (TR) of 9 ms, and an echo time (TE) of 4 ms. For the segmentation of MS-WML, also a 3D FLAIR sequence was acquired with a spatial resolution of 1.0 mm × 1.0 mm × 1.5 mm, a TR of 10^4^ ms, a TE of 140 ms, and a time to inversion of 2750 ms (Righart et al., 2017).

### Software

Lesions were segmented with the lesion growth algorithm (LGA, Schmidt et al., 2012) as implemented in the LST toolbox for SPM. The algorithm first segments the T1 images into the three main tissue classes cerebrospinal fluid (CSF), gray matter (GM) and WM. This information is then combined with the coregistered FLAIR intensities in order to calculate lesion belief maps. By thresholding these maps with a pre-chosen initial threshold (kappa), an initial binary lesion map is obtained which is subsequently grown along voxels that appear hyperintense in the FLAIR image. The result is a lesion probability map in MNI-space that can be thresholded to derive a MS-WML mask (MS-lesion voxels = 1, other voxels = 0). Data were acquired with the identical MRI scanner that was used for LGA development in 2012 and protocols also were exactly the same. Hence we worked with a kappa of 0.3, originally determined in 2012 by matching LST segmentation with expert manual segmentation. Empirical variograms of MS-WML masks were calculated with GSLIB (Deutsch and Journel, 1997). Variogram model fitting and longitudinal analysis were performed with R^1^, statistics on group age structures and results of variography and Gaussian Mixture Models were done with XLSTAT^2^. Voxler was used for MS-WML pattern visualization, 2D graphics were created with R and Grapher (Golden Software^3^).

#### Lesion Discrimination Plot (LDP, for Detailed Methodology See Marschallinger et al., 2016)

MS-WML pattern morphology was quantified by means of geostatistical variography. Geostatistics is a collection of algorithms for the analysis, modeling, and simulation of multidimensional data in a variety of disciplines (Caers, 2010). Variography is the central explorative data analysis (EDA) tool of geostatistics for measuring spatial correlation (Gringarten and Deutsch, 1999). The empirical variogram is calculated as follows (Eq. 1):

(1) $$\gamma (\text{h})=\frac{1}{2\text{n}(\text{h})}\cdot \sum_{\text{i}=1}^{\text{n}}{\left(\text{z}({\text{x}}_{\text{i}})-\text{z}({\text{x}}_{\text{i}}+\text{h})\right)}^{2}$$

Eq. 1: z(x) value of variable z at some 3D location x, here: voxel with z = binary variable (1 or 0); h lag vector of 3D separation between two relevant voxels (units: mm); n(h) number of voxel pairs [z(x), z(x+h)] at lag h; γ(h) empirical variogram value for lag h.

Computing, for a binary MS-WML pattern (i.e., a MS-WML mask with lesion voxels = 1, other voxels = 0), empirical variograms in x (left–right), y (posterior–anterior), and z (inferior–superior) directions, important aspects of the MS-WML pattern’s morphology are sensitively captured in terms of 3D correlation. Then, fitting a variogram model function (Cressie, 1993) to each of the x,y,z directional empirical variograms, the 3D morphology of the MS-WML pattern can be expressed by just two parameters per direction (x,y,z): the variogram range ***a*** and the variogram sill ***c***, i.e., ***a***_x_, ***a***_y_, ***a***_z_ and ***c***_x_, ***c***_y_, ***c***_z_. The exponential variogram model function (Eq. 2) was found suitable for modeling variograms of binary MS-WML patterns (Marschallinger et al., 2014).

(2) $$\gamma (\text{h})=\text{c}\cdot \left(1-\frac{\text{e}(-3\cdot |\text{h}|}{\text{a}}\right)$$

Eq. 2: c sill; a range; h lag vector of separation; *γ*(h) model variogram value for lag h.

**FIGURE 1:**
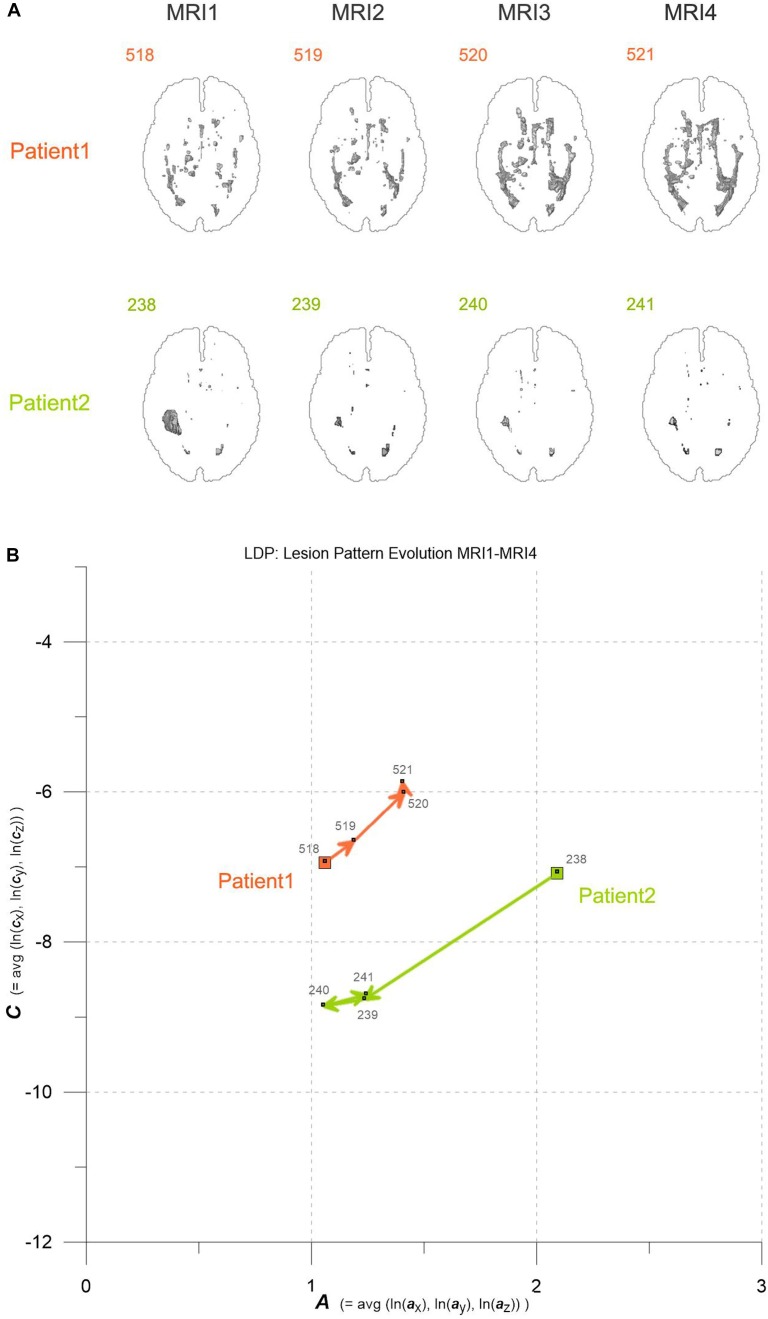
**(A)** Axial projections of evolving MS-WML patterns of two patients, documented by MRI1-MRI2-MRI3-MRI4. Numbers identify MS-WML patterns. Compare with Table 2 and **(B)**. **(B)** Abstraction of MS-WML pattern evolution in **(A)** to LDP framework. Numbers identify MS-WML patterns. Compare Table 2, see text for details.

**FIGURE 2:**
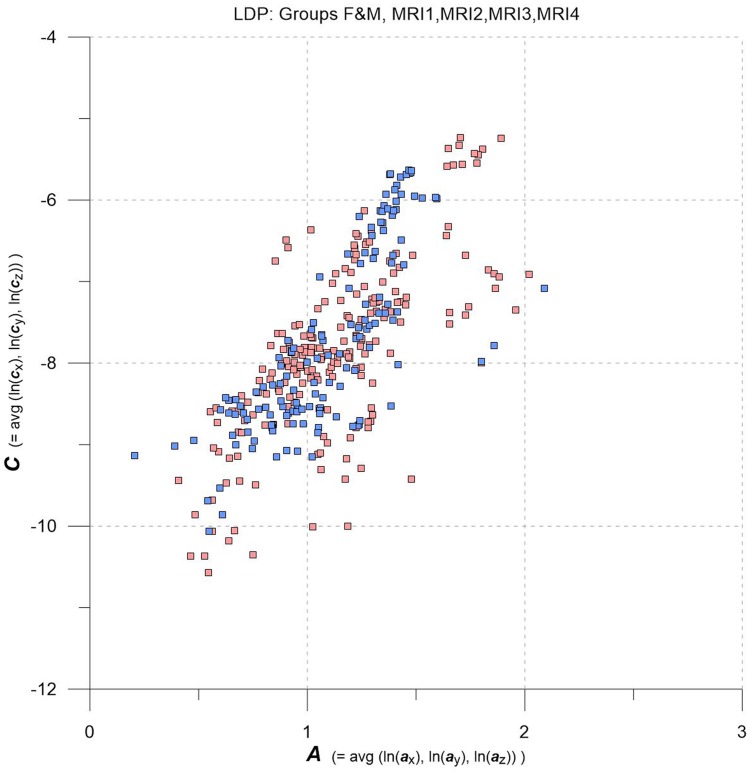
LDP with data from MRI1, MRI2, MRI3, MRI4, one square per MS-WML-pattern, stratified for groups F (red squares) and M (blue squares).

Variogram model function fitting was performed with R, yielding high-quality matching results with r^2^ typically 0.98. In consequence, derived parameters ***a*** and ***c*** are considered robust and significant. The variogram measures spatial correlation, which for binary patterns expresses spatial continuity. For 3D patterns, a high value of spatial correlation can be interpreted as surface smoothness while low spatial correlation points to surface complexness (Kourgli et al., 2004; Trevisani et al., 2009). The surface complexness of biological structures (like MS-WML patterns) is conveniently expressed as the ratio of surface area to enclosed volume (Schmidt-Nielson, 1984). For a binary MS-WML pattern, the variogram model range ***a*** is proxy of MS-WML pattern surface smoothness and the variogram model sill ***c*** is substitute of total lesion volume (TLV) (Marschallinger et al., 2016): the higher ***a***, the higher is the overall spatial correlation and the smoother (i.e., less geometrically complex) is the MS-WML pattern’s surface; the higher ***c***, the higher is TLV. Compare Figure 1A for examples – pattern 518 is “complex,” pattern 238 is “smooth.” The MS-Lesion Discrimination Plot (LDP) aims at mapping above 3D variography data to a dimension-reduced, well-arranged 2D space spanned by ***A*** on the abscissa, and ***C*** on the ordinate. For clarity of the LDP plot, each MS-WML pattern is displayed as a single point with ***A*** = avg(ln(***a***_x_), ln(***a***_y_), ln(***a***_z_)) and ***C*** = avg(ln(***c***_x_), ln(***c***_y_), ln(***c***_z_)). The advantage of logarithmic scaling is that MS-WML patterns with both mild and severe lesion load can be mapped in a single space (see Figures 1A, 2). When working with MS-WML patterns that are normalized to MNI space – recall that LGA results are already in MNI space – relative positions in the LDP can be interpreted in terms of DIS. Working with longitudinal data in MNI space, the LDP framework also enables straightforward graphical representation of MS-WML pattern evolution (DIT) in the individual MS disease course. This facilitates EDA of DIS and DIT, in single patient follow-up and intra- or inter-group data (Marschallinger et al., 2009, 2014).

## Results

As an example for practical LDP application, follow-up MRI data of two male patients (labeled “Patient1” and “Patient2,” both from group M) are contrasted in Figures 1A,B and in Table 2. Figure 1A shows axial projections of the MS-WML patterns derived from four successive MRIs (MRI1, MRI2, MRI3, MRI4) for Patient1 (patterns 518–521; 518 is an example of “complex pattern”) and Patient2 (patterns 238–241; 238 is an example of “smooth pattern”). Figure 1B gives the corresponding positions in the LDP; positions were connected by vectors to highlight patient-specific MS-WML pattern evolution paths. Since time lags between MRI exams are equal (1 year), the LDP also suggests dynamics of pattern geometry change – with long vectors signaling rapid change (due to logarithmic scaling of the LDP, visual matching of vector lengths and of evolution path directions is semi-quantitative only, however). Table 2 indicates that both patients start with similar TLV (at MRI1, Patient1: 4.90 cm^3^, Patient2: 4.05 cm^3^). In Figure 1A, MRI1 of Patient1 shows many scattered small- to medium-sized lesions while Patient2 has much of TLV accumulated in one big lesion. Progressing from MRI1 to MRI4, Patient1 shows steadily increasing TLV as well as increasing MS-WML pattern smoothness, which is due to lesion confluence. Most prominent changes occur between MRI2 and MRI3, ending up with a TLV of 9.16 cm^3^ at MRI4. As opposed, Patient2 shows decreasing TLV, mainly because the big lesion rapidly shrinks between MRI1 and MRI2. From MRI2 to MRI4, Patient2 MS-WML pattern remains visually almost unchanged with a final TLV of 0.61 cm^3^. Figure 1B illustrates the above context in the LDP. MS-WML pattern evolution of Patient1 is displayed with orange vectors, Patient2 with green vectors; numbers refer to MS-WML patterns in Figure 1A. In the LDP (Figure 1B), Patient1 and Patient2 evolution paths proceed in opposite directions. Patient1 vectors are generally pointing top right, indicating both increasing TLV and MS-WML pattern smoothness. The longest orange vector signals that prominent MS-WML pattern geometry changes occur between MRI2 and MRI3. By contrast, the evolution path of Patient2 MS-WML pattern is mainly running bottom left, with the longest green vector between MRI1 and MRI2. This is due to shrinkage of the big lesion and associated loss in TLV and loss in overall spatial correlation. Short vectors between MRI2-MRI3-MRI4 indicate only minor pattern changes occurring. The reversed vector direction between MRI3–MRI4 is consistent with two slightly increased lesions at posterior of pattern 241. Summing up, the LDP straightforwardly communicates different geometries, converse evolutions and dynamics of Patient1 and Patient2 MS-WML patterns.

**Table 2:** MS-WML pattern evolution of two MS-patients: variogram model parameters and total lesion volume (TLV) derived from MRI1 and MRI4.

		MRI1			MRI4		
	*A*	*C*	TLV	*A*	*C*	TLV
Patient1	1.06	−6.94	4.90	1.40	−5.87	9.16
Patient2	2.09	−7.08	4.05	1.24	−8.70	0.61

*Compare with Figures 1A,B. **A** = avg(ln(**a**_x_), ln(**a**_y_), ln(**a**_z_)) and **C** = avg(ln(**c**_x_), ln(**c**_y_), ln(**c**_z_)). See text for details.*

**FIGURE 3:**
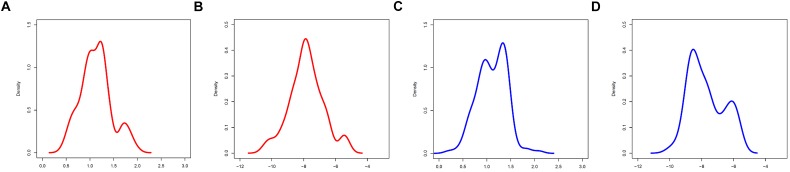
Marginal ***A,C*** distributions of groups F (red) and M (blue). **(A,B) *A,C*** group F. **(C,D) *A,C*** group M.

**FIGURE 4:**
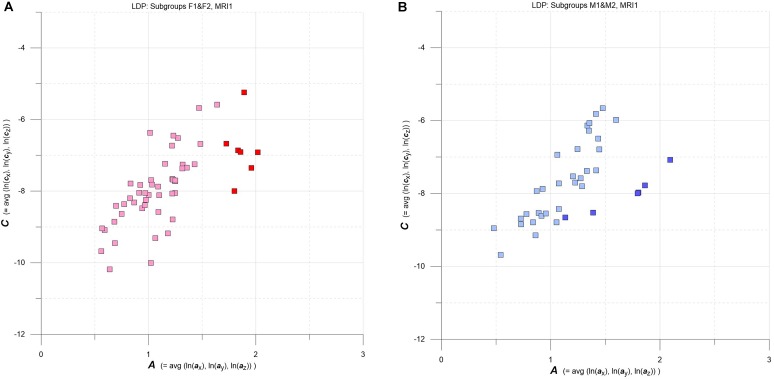
**(A,B)** LDPs with F and M subgroups derived by GMM clustering. **(A)** Subgroups F1 and F2 (F1: pink squares, *n* = 46, average maximum a posteriori probability of class membership (MAP) = 0.992; F2: red squares, *n* = 7, average MAP = 0.911). **(B)** Subgroups M1 and M2 (M1: light blue squares, *n* = 30, average MAP = 0.989; F2: blue squares, *n* = 6, average MAP = 0.909). See text for details.

As an overview, the LDP in Figure 2 depicts all MS-WML patterns extracted from MRI1, MRI2, MRI3, and MRI4. The plot is stratified for groups F (red squares) and M (blue squares). At first glimpse, both groups display as strongly overlapping, elliptic point-clouds with an imaginary long axis trending 45 degrees, from about the lower left to the upper right corners of the LDP. Point densities are highest at and around the imaginary long axis, with a gentle fall-off toward both ends of the long axis. There is also a fall-off in F and M point densities perpendicular to the long axis, more distinct toward top left and dispersed toward bottom right. Closer inspection of Figure 2 reveals that F points markedly overbalance M points along the lower fringe of the point cloud. In the LDP, MS-WML patterns that plot near the lower fringe of the cloud typically are dominated by relatively few, extended lesions while patterns that plot near the upper fringe of the point cloud are characterized by many small lesions or complexly shaped MS-WML aggregates (Marschallinger et al., 2016; also check pattern 238 against pattern 518 in Figure 1A).

Tentatively, MS-WML patterns that are dominated by continuous, extended lesions should be more abundant in group F than in group M. To overcome the inherent limitations of visually interpreting MS-WML point-clouds in the LDP (Figure 2), we reviewed the marginal distributions with density plots. Figure 3 suggests that both F and M groups have multimodal ***A*** and ***C*** distributions.

Going into more detail, we used multidimensional Gaussian Mixture Modeling (GMM) on ***A***, ***C*** data extracted at baseline (MRI1) to check groups F and M for possible clustering in the LDP space. Results are contrasted in Figures 4A,B.

Interestingly, at baseline GMM yields comparable clustering results for both F and M groups: a larger subgroup that stretches approximately along the diagonal of the LDP and a smaller subgroup that plots at comparatively high levels of ***A*** and ***C***. The larger subgroups are tagged F1 (pink) and M1 (light blue), and the smaller subgroups are F2 (red) and M2 (blue).

Normality tests (Shapiro–Wilk, Lilliefors, Anderson-Darling, all with α = 0.05) on the respective marginal ***A*** and ***C*** distributions gave ambiguous results. Therefore, the evolution of ***A*** and ***C*** distributions in the above subgroups, from MRI1 (baseline) to MRI4 (end of study), is portrayed with box-whsiker plots (Chambers et al., 1983). The relevant F1, F2, M1, M2 distributions were matched on a non-parametric basis with robust statistical tests [Kruskal–Wallis (KW) and Mann–Whitney (MW) tests, all with α = 0.05].

**FIGURE 5:**
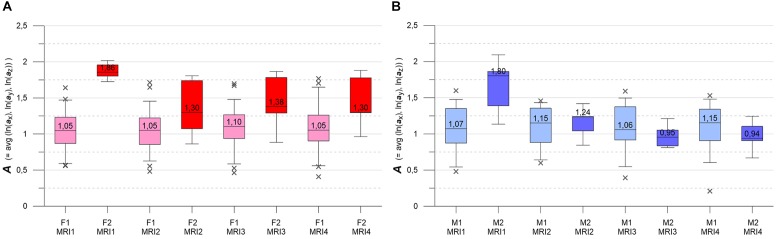
**(A,B)** Box-whisker plots indicating longitudinal evolution of ***A*** distributions, from MRI1 to MRI4. Box height is interquartile range (IQR), line with value is median, whiskers mark 5 and 95% quantiles, crosses are outliers. (**A**, left) Subgroups F1 (pink, *n* = 46) and F2 (red, *n* = 7). (**B,** right) Subgroups M1 (light blue, *n* = 30) and M2 (blue, *n* = 6). See text for details.

**FIGURE 6:**
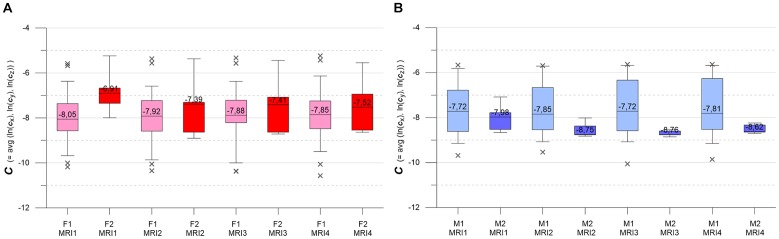
**(A,B)** Box-whisker plots indicating longitudinal evolution of ***C*** distributions, from MRI1 to MRI4. (**A**, left) Subgroups F1 (pink, *n* = 46) and F2 (red, *n* = 7). Figure 5B, right: subgroups M1 (light blue, *n* = 30) and M2 (blue, *n* = 6). Graphics parameters as in Figures 5A,B, see text for details.

Figures 5A,B contrast the longitudinal evolution of the ***A*** distributions of subgroups F1, F2, M1, M2 at MRI1, MRI2, MRI4, MRI4. Aside from minor fluctuations, F1 and M1 show nearly constant longitudinal medians, IQRs and whiskers. Accordingly, KW- and MW-tests do not indicate significant differences for the longitudinal ***A*** distributions in F1 and M1. F2 and M2 present more heterogeneously, with pronounced variations in medians, IQRs and whiskers. At baseline, both F2 and M2 start with high-level medians that decrease strongly at MRI2, followed by about constant values in F2 and a further decrease in M2. While F2 medians clearly remain higher than the respective F1 medians, M2 medians sink below M1 medians at MRI3. For both F2 and M2, KW-tests yield significant longitudinal differences. At all MRIs, MW-tests show significant differences between subgroups F1 and F2. MW-tests also show significant differences between M1 and M2 at MRI1 and between F2 and M2 at MRI3, MRI4.

Figures 6A,B present the longitudinal development of ***C*** distributions in subgroups F1, F2, M1, M2 at MRI1, MRI2, MRI3, MRI4. F1 and M1 show only minor longitudinal variation in medians, IQRs and whiskers, but F1 has a tendency of slightly increasing medians. KW- and MW-tests do not indicate significant differences for longitudinal ***C*** distributions in F1, M1. As compared to F1 and M1, F2 and M2 show accented variations in medians, IQRs and whiskers. F2 medians are longitudinally sinking but remain higher than respective F1 medians. M2 medians are also sinking (exception: MRI4) but are clearly lower than the respective M1 medians. MW-tests show significant differences between F1, F2 and F2, M2 at MRI1 and between M1, M2 at MRI2, MRI3, MRI4.

**FIGURE 7:**
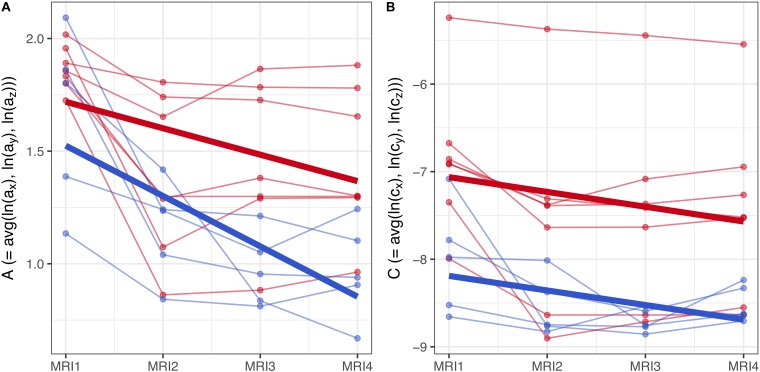
**(A,B)** Longitudinal analysis of geostatistical parameters ***A*** and ***C*** in subgroups F2 and M2, from MRI1 to MRI4. See text for details.

To assess the statistical significance of gender-based differences in the evolution of parameters ***A*** and ***C*** in groups F2 and M2, a longitudinal analysis was performed. A linear mixed model with fixed effects for centered time-point, gender and their interaction was used to estimate the group-level differences between genders. Subject-specific variation was accounted for by random effects for the intercept and centered time-point. Results are summarized graphically in Figures 7A,B: thin lines indicate the subject-specific evolution of parameters ***A*** and ***C*** for group F2 (red) and M2 (blue) individuals, from MRI1 to MRI4. Thick lines refer to the group-level change as estimated by the fixed effects – they indicate negative slopes for ***A*** and ***C*** in both groups F2, M2 without significant difference as well as a well-discernible offset (expected diff. between M2 and F2 between MRI2 and MRI3: −0.35 (95% CI: [−0.63, −0.08]) for parameter ***A***; −1.12 (95% CI: [−2.04, −0.21]) for parameter ***C***).

Summing up, longitudinal analysis results are in accordance with EDA: in subgroups F2 and M2, females show significantly higher values of ***A*** and ***C*** than males.

**FIGURE 8:**
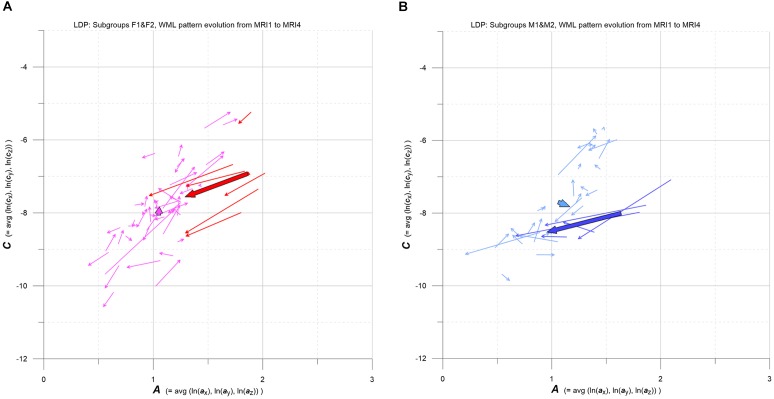
**(A,B)** LDP-based MS-WML pattern evolution plot. Vectors indicate F1 (pink), F2 (red), M1 (light blue), and M2 (blue) individual and subgroup evolution in the 3-year observation period, from MRI1 to MRI4. One thin vector per individual, total vectors are bold, based on relevant medians. See text for details.

As pointed out in Figure 1B, the evolution of MS-WML patterns can be conveniently visualized in the LDP by connecting longitudinal ***A***,***C*** data with vectors (Figure 8). Since the individual MS-WML patterns of F and M groups are in MNI geometry and time between MRI1 and MRI4 is 3 years, the dynamics of pattern change can be visually checked.

Figure 8 combines two LDPs that summarize individual-based and subgroup-based longitudinal MS-WML pattern evolution from baseline (MRI1) to study end (MRI4). Figure 8A shows subgroups F1 (pink) and F2 (red), Figure 8B shows subgroups M1 (light blue) and M2 (blue). Vectors start at MRI1, arrow heads point to MRI4. Individual vectors are thin, subgroup total vectors are bold. Per subgroup, total vectors are defined by spatial medians of ***A***,***C*** at MRI1 and MRI4 (compare Figure 5 and Figure 6). Individual vectors of subgroups F1, M1 roughly start along the LDP diagonal and show mixed directions and magnitudes. In consequence, total vectors of subgroups F1, M1 start at medium ***A***,***C*** levels and have minor magnitudes, indicating negligible changes: F1 points toward minimally increased ***A*** and ***C***, M1 points toward slightly increased ***A*** and decreased ***C***. By contrast, individual vectors of subgroups F2, M2 start at high ***A*** and medium ***C*** levels, and are mostly pointing to strongly decreased ***A***, and to less decreased ***C***. Hence F2 and M2 total vectors start at high ***A*** and medium ***C***, have increased magnitudes and face bottom-left. Notably, F2 starts and ends at clearly higher ***A***,***C*** than M2.

**Table 3:** Relations between subgroup ***A*** and EDSS at MRI1 and MRI4.

	MRI1		MRI4			
	*A*	EDSS	*A*	EDSS	ΔA	ΔEDSS
F1	1.05	1.4	1.05	1.5	0.00	0.1
F2	1.86	0.9	1.30	0.6	−0.56	−0.3
M1	1.07	1.2	1.15	1.5	0.08	0.3
M2	1.80	1.0	0.94	0.5	−0.86	−0.5

*[ΔA = A(MRI4)-A(MRI1), ΔEDSS = EDSS(MRI4)-EDSS(MRI1)]. See text for details.*

Subgroup average EDSS deltas broadly correspond to subgroup ***A*** deltas (Table 3): from MRI1 to MRI4, subgroups F1 and M1 show no/minor increase in ***A*** and slightly increased average EDSS, while subgroups F2 and M2 present clearly reduced ***A*** and reduced average EDSS.

## Discussion

The aim of this study was to evaluate gender-related MS-WML pattern morphology evolution in early MS. Using demographically comparable groups, MRI data acquired by standardized methodology and automatic MS-WML extraction, geostatistical variography revealed differences in the spatiotemporal evolution of MS-WML patterns among women and men with early-MS. The geostatistical work flow for quantifying MS-WML patterns is well-established and provides reliable measures on spatial correlation and TLV. Variography parameters ***A***,***C*** are considered appropriate for reducing the potentially complex three-dimensional structures of MS-WML patterns to two dimensions, for representing DIS in coherent form in the LDP.

We could use the LDP as an EDA tool for revealing the following differences in DIS and DIT: corresponding to gender and MS-WML pattern morphology at baseline, two female subgroups (F1, F2) and two male subgroups (M1, M2) are discerned. Subgroups F1, M1 and F2, M2 show clearly different evolution in space and time. At baseline, both F1 and M1 show similar, medium-level ***A***,***C*** values that remain practically unchanged till end of study – total vectors in the LDP indicate quasi-stationary behavior of F1, M1. By contrast, subgroups F2 and M2 show pronounced variations in the LDP: at baseline, both subgroups start with high-level ***A*** and medium-level ***C***, and evolve toward markedly lower ***A***,***C*** values at end of study. Both M2 and F2 total vectors indicate distinctly reduced pattern smoothness and TLV. Compared to M2, the F2 total vector starts and ends at significantly higher ***A***,***C*** levels, however.

The dominant physical equivalent of increasing parameters ***A*** and ***C*** is lesion growth or confluence while decreasing ***A*** and ***C*** point to shrinking lesions or to breakup of lesion aggregates. Given MS-WML occur at preferred locations (Filli et al., 2012), notably alongside the CSF system which is stretched in y and z directions, MS-WML patterns are expected to show increased spatial correlation in these directions. Separate examination of x,y,z components of ***A*** complies with this expectation, in all subgroups. Generally, when matching total vectors in the MS-WML pattern evolution plot, in the 3-years observation period subgroups F1 and M1 show practically constant, medium-level MS-WML pattern smoothness and TLV, i.e., negligible change on average. In contrast, F2 and M2 that start with high-level MS-WML pattern smoothness and TLV, are characterized by strongly diminishing MS-WML pattern smoothness and reducing TLV – i.e., continued shrinking and breakup of MS-WML. From baseline to end of study, MS-WML patterns with spatially highly correlated, smooth lesions remain more abundant in F2 than in M2.

Striving for an interpretation of above results, group characteristics are recalled: groups studied here are early-MS with DMD and less than 2 years from first MS signs to MRI1. Of note, Frischer et al. (2015) describe that active WM MS plaques predominate in acute and early MS pathology. This could explain part of the MS-WML morphology evolution in F2 and M2, where dominant ***A*** and ***C*** reduction is observed from MRI1 to MRI2. Dunn et al. (2015) state that the autoimmune component which predominates in early-stage MS is more robust in women than in men and Antulov et al. (2009) show that WM atrophy is more advanced in female patients – this might account for the higher ***A*** and ***C*** values of F2 as compared to M2, over the whole 3-year observation period. Additional reasons for this phenomenon could be a distinct remyelination activity and presence of lesions with differing extent of activity among sexes. In this regard, a histopathological analysis by Kuhlmann et al. (2009) found that remyelination is slightly, but not significantly, more extensive in women than men in early MS lesions. A more recent study by Luchetti et al. (2018) reported a higher proportion of mixed active/inactive lesions compared to females. Last no least, the distinct reduction of ***A*** and ***C*** in F2 and M2 subgroups could relate to a stronger response to DMD of patients with smoother MS-WML. Interestingly, data on subgroup parameter ***A*** respectively EDSS at baseline and at end of study suggest a positive correlation. One problem in relating ***A*** and EDSS is the fact that ***A*** measures brain MS-WML pattern morphology only, while EDSS combines brain and spine performance. This might introduce unknown bias. A limitation of the current approach to MS-WML pattern dynamics is that it relies on reproducible MRI input data. For example, matching longitudinal data involving different MRI equipment and varying MS-WML extraction methods, we could not find significant gender differences in MS-WML patterns. While the inherent strength of spatial summary statistics on MS-WML-patterns provided by variography is to communicate the “broad picture,” this is counterbalanced by loss of spatial granularity: variography is not sensitive to location. In this respect, if more spatial granularity is necessary, MS-WML patterns need to be confined by ROIs. In a next step, these first explorative results on a sexually bimorphic evolution of MS-WML-patterns need to be verified with larger data sets in order to better quantify the uncertainties of estimates.

## Ethics Statement

This study was carried out in accordance with the recommendations of Ethikkommission Land Salzburg. The protocol was approved by the Ethikkommission Land Salzburg. As this study involves only retrospective data and all subjects were anonymized before processing, the study was exempt from a detailed ethics statement (see translation below). Translation: “Research Projects that do not include clinical exams as by the Drug Law, Medical Products Law or new medical methods including non-interventional studies or applied medical research as by the Salzburg Clinics Law are not subject to evaluation by the Federal Country of Salzburg Ethics Committee” (Original in German).

## Author Contributions

RM, MM, and JS contributed to the conception and design of the study. VP and JK organized the database. RM performed geostatistical analysis. RM and JS wrote the first draft of the manuscript. MM wrote sections of the manuscript. PS performed longitudinal statistics and helped with general statistics. SM performed longitudinal MS-WML extraction. All authors contributed to manuscript revision, read and approved the submitted version.

## Conflict of Interest Statement

The authors declare that the research was conducted in the absence of any commercial or financial relationships that could be construed as a potential conflict of interest.

## Footnotes

1 https://cran.r-project.org

2 https://www.xlstat.com

3 http://www.goldensoftware.com

## References

[B1] Antulov R. Weinstock-Guttman B. Cox J. L. Hussein S. Durfee J. (2009). Gender-related differences in MS: a study of conventional and nonconventional MRI measures. *Mult. Scler.* 15 345–354. 10.1177/1352458508099479 19028830

[B2] Caers J. (2010). *Modeling Uncertainty in the Earth Sciences.* Hoboken, NJ: Wiley-Blackwell, 246.

[B3] Chambers J. M. Cleveland W. S. Kleiner B. Tukey P. A. (1983). “Comparing data distributions,” in *Graphical Methods for Data Analysis*, eds Breiman L. Breiman L. (Belmont, CA: Wadsworth International Group), 62.

[B4] Cressie N. A. C. (1993). *Statistics for Spatial Data.* Hoboken, NJ: Wiley, 920.

[B5] Deutsch C. V. Journel A. G. (1997). *GSLIB Geostatistical Software Library and User’s Guide.* New York, NY: Oxford University Press, 380.

[B6] Dolezal O. Gabelic T. Horakova D. Bergsland N. Dwyer M. G. Zivadinov R. (2013). Development of gray matter atrophy in relapsing-remitting multiple sclerosis is not gender dependent: results of a 5-year follow-up study. *Clin. Neurol. Neurosurg.* 115(Suppl. 1)), 42–48. 10.1016/j.clineuro.2013.09.020 24321154

[B7] Dunn S. E. Lee H. Pavri F. R. Zhang M. A. (2015). Sex-based differences in multiple sclerosis (Part I): biology of disease incidence. *Curr. Top. Behav. Neurosci.* 26 29–56. 10.1007/7854-2015-371 25690593

[B8] Filli L. Hofstetter L. Kuster P. Bendfeldt K. (2012). Spatiotemporal distribution of white matter lesions in relapsing-remitting and secondary progressive multiple sclerosis. *Mult. Scler.* 18 1577–1584. 10.1177/1352458512442756 22495945

[B9] Frischer J. M. Weigand S. D. Guo Y. Kale N. Parisi J. E. Luccincetti C. (2015). Clinical and pathological insights into the dynamic nature of the white matter multiple sclerosis plaque. *Ann. Neurol.* 78 710–721. 10.1002/ana.24497 26239536 PMC4623970

[B10] Gringarten E. Deutsch C. V. (1999). Methodology for variogram interpretation and modeling for improved reservoir characterization. *Proc. SPE* 56654 1–13. 10.2118/56654-MS

[B11] Harbo H. F. Gold R. Tintoré M. (2013). Sex and gender issues in multiple sclerosis. *Ther. Adv. Neurol. Disord.* 6 237–248. 10.1177/1756285613488434 23858327 PMC3707353

[B12] Kalincik T. Vivek V. Jokubaitis V. Lechner-Scott J. Trojano M. Izquierdo G. (2013). Sex as a determinant of relapse incidence and progressive course of multiple sclerosis. *Brain* 136(Pt 12), 3609–3617. 10.1093/brain/awt281 24142147

[B13] Kourgli A. Belhadj-aissa A. Bouchemakh A. (2004). “Optimizing texture primitives description based on variography and mathematical morphology,” in *Image Analysis and Recognition*, eds Campilho A. Kamel M. (Toronto, ON: ICIAR), 862.

[B14] Kuhlmann T. Goldschmidt T. Antel J. Wegner C. König F. Metz I. (2009). Gender differences in the histopathology of MS? *J. Neurol. Sci.* 286 86–91. 10.1016/j.jns.2009.07.014 19674757

[B15] Louapre C. (2018). Conventional and advanced MRI in multiple sclerosis. *Rev. Neurol.* 174 391–397. 10.1016/j.neurol.2018.03.009 29784248

[B16] Luchetti S. Fransen N. L. van Eden C. G. Ramaglia V. Mason M. Huitinga I. (2018). Progressive multiple sclerosis patients show substantial lesion activity that correlates with clinical disease severity and sex: a retrospective autopsy group analysis. *Acta Neuropathol.* 135 511–528. 10.1007/s00401-018-1818-y 29441412 PMC5978927

[B17] Marschallinger R. Golaszewski S. M. Kraus J. Kronbichler M. Kunz A. Hofmann P. (2009). “Multiple sclerosis: a multidisciplinary approach to the analysis, 4D modeling and spatiotemporal simulation of lesion pattern evolution,” in *Proceedings of the 4th SEECCM*, eds Papadrakakis M. Kojic M. Papadopoulos V. (Kragujevac).

[B18] Marschallinger R. Golaszewski S. M. Kunz A. B. Kronbichler M. Ladurner G. Kraus J. (2014). Usability and potential of geostatistics for spatial discrimination of multiple sclerosis lesion patterns. *J. Neuroimaging* 4 278–286. 10.1111/jon.12000 23384318

[B19] Marschallinger R. Schmidt P. Hofmann P. Zimmer C. Atkinson P. M. Sellner J. (2016). A MS-lesion pattern discrimination plot based on geostatistics. *Brain Behav.* 6:e00430. 10.1002/brb3.430 26855827 PMC4733107

[B20] Righart R. Biberacher V. Jonkman L. E. Klaver R. Schmidt P. Mühlau M. (2017). Cortical pathology in multiple sclerosis detected by the T1/T2-weighted ratio from routine magnetic resonance imaging. *Ann. Neurol.* 82 519–529. 10.1002/ana.25020 28833433 PMC5698772

[B21] Schmidt P. Gaser C. Arsic M. Buck D. Forschler A. (2012). An automated tool for detection of FLAIR-hyperintense white-matter lesions in multiple sclerosis. *NeuroImage* 59 3774–3783. 10.1016/j.neuroimage.2011.11.032 22119648

[B22] Schmidt-Nielson K. (1984). *Scaling: Why is Animal Size So Important?* Cambridge: Cambridge University Press, 237.

[B23] Schoonheim M. M. (2014). Sex-specific extent and severity of white matter damage in multiple sclerosis: implications for cognitive decline. *Hum. Brain Mapp.* 35 2348–2358. 10.1002/hbm.22332 23982918 PMC6869647

[B24] Shirani A. Zhao Y. Kingwell E. Fisk J. Rieckmann P. Tremlett H. (2012). Temporal trends of disability progression in multiple sclerosis: findings from British Columbia, Canada (1975-2009). *Mult. Scler* 18 442–450. 10.1177/1352458511422097 21952097

[B25] Thompson A. J. Banwell B. L. Barkhof F. Carroll W. M. Coetzee T. Cohen J. A. (2018). Diagnosis of multiple sclerosis: 2017 revisions of the McDonald criteria. *Lancet Neurol.* 17 162–173. 10.1016/S1474-4422(17)30470-2 29275977

[B26] Tomassini V. Pozzilli C. (2009). Sex hormones, brain damage and clinical course of multiple sclerosis. *J. Neurol. Sci.* 286 35–39. 10.1016/j.jns.2009.04.014 19426994

[B27] Trevisani S. Cavalli M. Marchi L. (2009). Variogram maps from LiDAR data as fingerprints of surface morphology on scree slopes. *Nat. Hazards Earth Syst. Sci.* 9 129–133. 10.5194/nhess-9-129-2009

